# Subcutaneous Implantable Cardioverter Defibrillator: Early Experience

**DOI:** 10.21470/1678-9741-2017-0082

**Published:** 2017

**Authors:** Fernando Sérgio Oliva Souza, Vanessa Sepulvida Matos, Marcos Cesar Valério Almeida, Samuel Campagiotto Weiss, Lucas Henrique Borges Rodrigues, Pedro Augusto Gori Lima, Davi Bongiolo Mattos

**Affiliations:** 1 Instituto de Arritmias Cardíacas (IAC), São Paulo, SP, Brazil.; 2 Hospital Beneficência Portuguesa São Paulo, São Paulo, SP, Brazil.

**Keywords:** Death, Sudden, Cardiac, Defibrillators, Implantable, Arrhythmias, Cardiac/therapy

## Abstract

**Introduction:**

The implantable cardioverter defibrillator had been increasing the survival of patients at high risk for sudden cardiac death. The subcutaneous implantable cardioverter defibrillator was developed to mitigate the complications inherent to lead placement into cardiovascular system.

**Objective:**

To report the initial experience of 18 consecutive cases of subcutaneous implantable cardioverter defibrillator implantation showing the indications, potential pitfalls and perioperative complications.

**Methods:**

Between September 2016 and March 2017, 18 patients with indication for primary and secondary prevention of sudden cardiac death, with no concomitant indication for artificial cardiac pacing, were included.

**Results:**

The implantation of the subcutaneous implantable cardioverter defibrillator successfully performed in 18 patients. It was difficult to place the subcutaneous lead at the parasternal line in two patients. One patient returned a week after the procedure complaining about an increase in pain intensity at pulse generator pocket site, which was associated with edema, temperature rising and hyperemia. Two patients took antialgic medication for five days after surgery. A reintervention was necessary in one patient to replace the lead in order to correct inappropriate shocks caused by myopotential oversensing.

**Conclusion:**

In our initial experience, although the subcutaneous implantable cardioverter defibrillator implantation is a less-invasive, simple-accomplishment procedure, it resulted in a bloodier surgery perhaps requiring an operative care different from the conventional. Inappropriate shock by oversensing is a reality in this system, which should be overcame in order not to become a limiting issue for its indication.

**Table:** 

Abbreviations, acronyms & symbols	
ATP	= Antitachycardia pacing
BMI	= Body Mass Index
DFT	= Defibrillation test
ICD	= Implantable Cardioverter Defibrillator
NSAIDs	= Non-steroidal anti-inflammatory drugs
SCD	= Sudden cardiac death
S-ICD	= Subcutaneous implantable cardioverter defibrillator
VF	= Ventricular fibrillation

## INTRODUCTION

The implantable cardioverter defibrillator (ICD) has been increasing the survival of patients at high risk for sudden cardiac death (SCD)^[1,2]^. Advancements in programming this device have been significantly decreased the necessity for shocks and their quantity, otherwise, acute and chronic complications related to the implantation of a transvenous ICD (TV-ICD) result in a significant increase in peri-operative morbidity^[3]^.

Currently, the insertion of leads into the central venous system and within cardiac chambers is considered the most fragile portion of this pacing system since it could cause vascular obstruction, thrombosis, cardiac perforation and cardiac tamponade; leads are also associated with infectious complications, such as endocarditis, and mechanical complications, such as pneumothorax^[4,5]^. Silicone breakage, micro-fracture or even the fracture of the lead, which is estimated in 0.58% per year and more than 20% in 10 years^[6,7]^ could lead to inappropriate shocks or non-delivery of appropriate shocks.

The subcutaneous implantable cardioverter defibrillator (S-ICD) was developed to mitigate the complications inherent to lead placement into cardiovascular system aiming primarily patients with congenital heart disease, immunosuppressed patients, patients with permanent intravascular access for hemodialysis, or those with severe cardiovascular system problems in which lead passage would be almost impossible. The result was the development of a device whose lead preserves the cardiovascular system. The S-ICD was authorized in Europe in 2009, in the United States in 2012 and recently this new technology has been authorized in Brazil as well.

The purpose of this study is to report the initial experience of 18 consecutive cases of S-ICD implantation showing the indications and peri-operative complications.

## METHODS

**Fig. 1:**
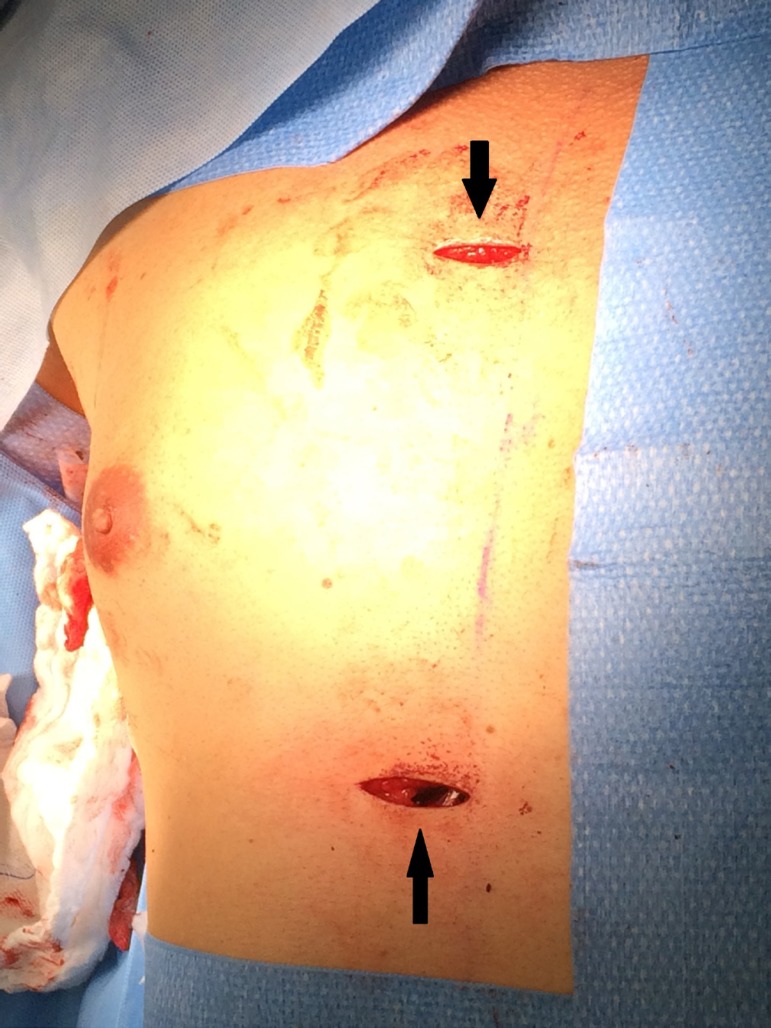
The picture is showing the two last incisions. The upper arrow in pointing out the last incision and the lower arrow is pointing out the second incision.

**Fig. 2:**
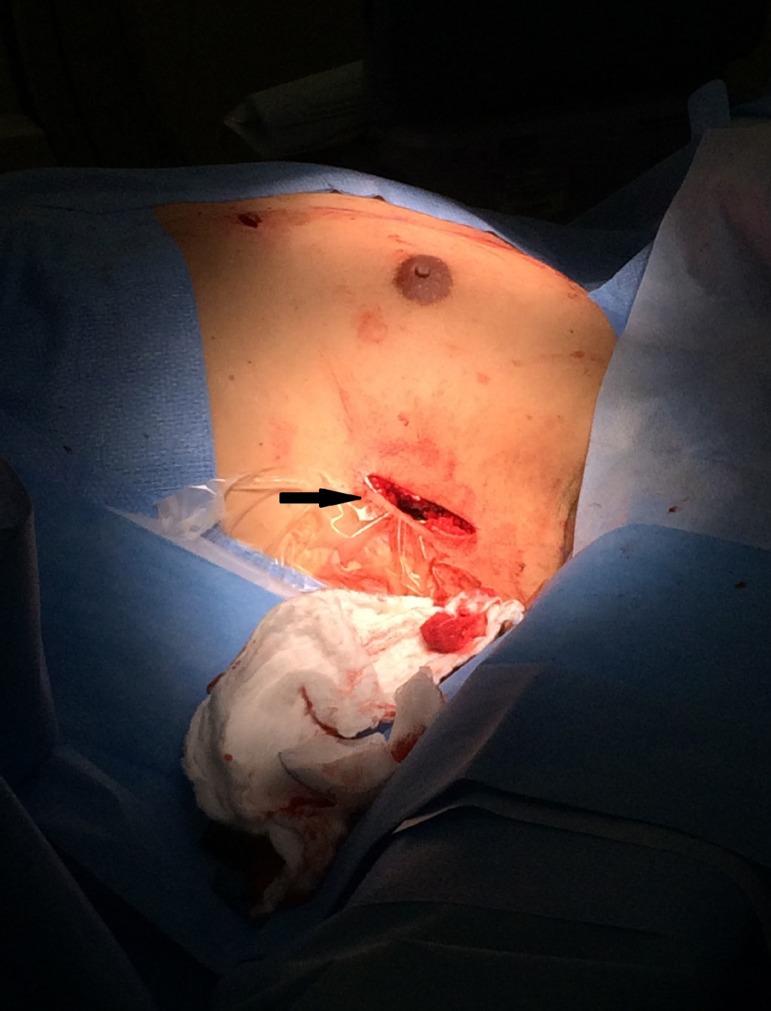
The picture is showing the left lateral thorax view. The arrow is pointing out the first incision.

**Fig. 3:**
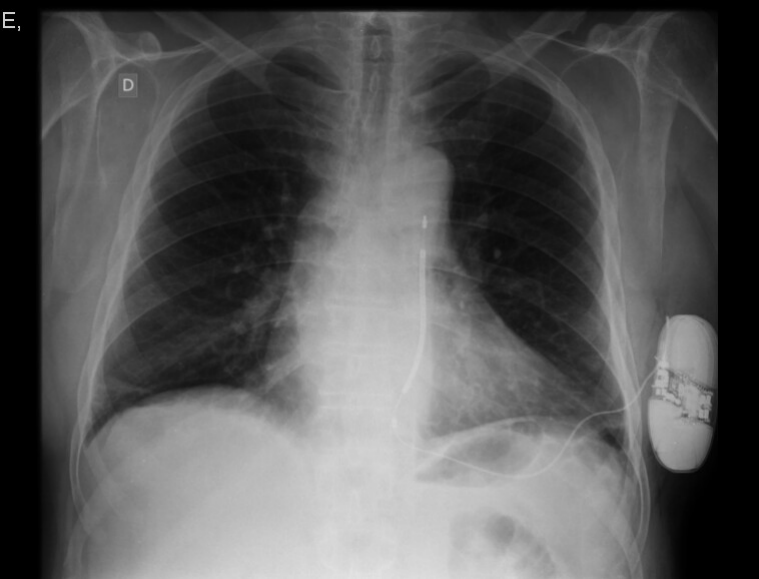
Postoperative posteroanterior (PA) chest radiograph

Between September 2016 and March 2017, 18 patients with indication for primary and secondary prevention of SCD, with no concomitant indication for artificial cardiac pacing for the treatment of symptomatic bradycardia, cardiac resynchronization therapy and/or antitachycardia therapy, were included. After S-ICD potential benefits had been presented to the patients and accepted by them, the patients were submitted to the screening phase by validating the QRS complexes through a specific ruler designed for this purpose. The procedure was performed in the operating room under general anesthesia. After antisepsis and surgical fields positioning, three incisions were made in the first 15 patients and just two incisions in the last three patients. The first one to create the pocket in which the pulse generator will be placed was made with approximately 6 cm between the fifth and sixth intercostal spaces, nearby the nipple, beginning next to the anterior axillary line towards the middle axillary line; the second one with 3 cm in parasternal position nearby the xiphoid process and the third one with 2 cm at the third intercostal space region (Figures 1 and 2). The lead was tunneled through the first incision, made to create the pocket, to the incision next to the xiphoid process and then to the third incision, approximately 14 cm in straight line to sternal, at the third space region. All these tunnelizations were performed between the muscle plan and the subcutaneous tissue. Afterwards, the lead was connected to the pulse generator, which was positioned in the pocket made between the fascia of the transverse muscle and the great dorsal muscle (Figure 3). The last three patients had the lead tunneled from the second incision toward to the Louis angle with a 11 French introducer avoiding the third incision. Then, the defibrillation test (DFT) was performed in order to assess the system efficiency.

## RESULTS

**Fig. 4:**
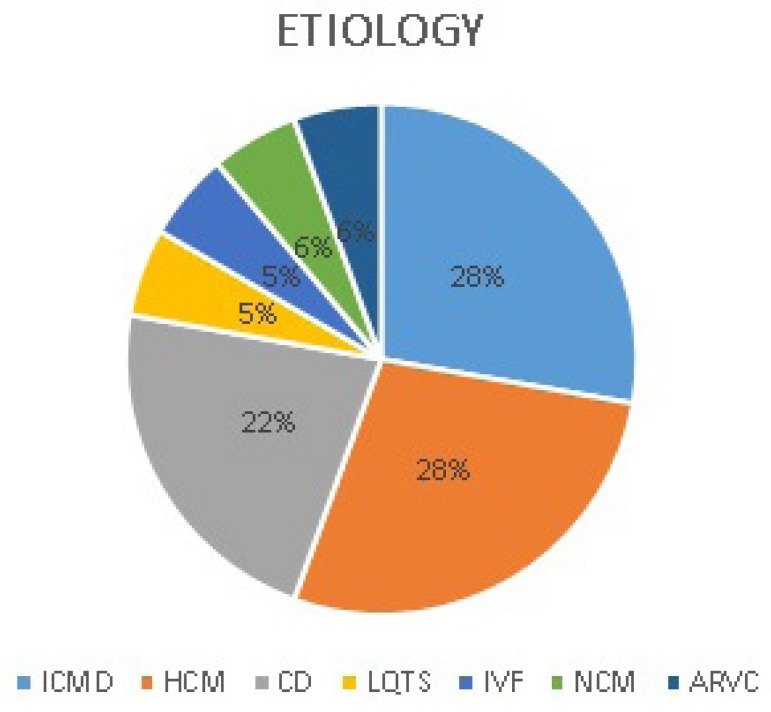
Frequency and etiologyfor the S-ICD. HCM=hypertrophic cardiomyopathy; ICMD=dilated ischemic cardiomyopathy; CD=Chagas disease; ARVC=arrhythmogenic right ventricular cardiomyopathy; LQTS=congenital long QT; NCM=non-compacted myocardium, IVF=primary ventricular fibrillation

The implantation of the S-ICD was successfully performed in the 18 patients, four female and 15 male, with mean age 52±12 years, and mean EF 48±11.8% (Figures 3 and 4). Four patients had indication for secondary prevention and 14 for primary prevention. Five out of 18 patients had hypertrophic cardiomyopathy, five with ischemic cardiomyopathy, four with Chagas disease cardiomyopathy, one with non-compacted myocardium, one with arrhythmogenic right ventricular cardiomyopathy, one with primary ventricular fibrillation (VF) and one with congenital long QT syndrome (Figure 4).

It was difficult to place the subcutaneous lead at the parasternal line in two patients, one due to grade II obesity with body mass index (BMI) of 32 and the other one due to *pectus excavatum* thoracic deformity. In all cases the cut off heart rate was set up to 170 beats per minute. In 16 cases, VF was induced and reverted to sinusal rhythm with a 65 Joules shock. In one case, even after the repositioning of the electrode, a successfully defibrillation only occurred after the second shock with 80 Joules and, in another patient, even with an appropriate positioning, the sinus rhythm was only restored with the third shock, both patients were considered to a late retest for at least 30 days after the procedure. The mean time between sensing and shock was 18±3 seconds.

One patient returned a week after the procedure complaining about an increase in pain intensity at pulse generator pocket site, which was associated with edema, temperature rising and hyperemia. This clinical condition was solved providing nonsteroidal anti-inflammatory drugs and therapy with antibiotics over 7 days and two patients demanded antialgic medication for five days after the procedure. A reintervention was necessary in one patient to replace the lead in order to correct inappropriate shocks caused by myopotential oversensing.

## DISCUSSION

The greatest advantage of the S-ICD is avoiding the implantation of leads within cardiovascular system, thus preserving central venous circulation, with no risks of mechanical traumas, such as vascular damages or pneumothorax, and with a very low risk of systemic infection^[8]^. An important disadvantage is the impossibility of the system to provide heart pacing. For this reason, it is contraindicated for patients with indication for bradycardia pacing, cardiac resynchronization therapy or antitachycardia pacing (ATP) since S-ICD cannot provide cardiac pacing other than for a short period post-shock, when necessary^[9]^. Based on the benefits and after pre-selecting the patients, this new technology was chosen to be used.

After selecting the patients, they were submitted to the screening phase using a customized plastic ruler supplied by the manufacturer with the purpose of evaluating if the generated electric signal would be able to provide an appropriate functioning of the S-ICD. Although it is estimated that 7% of the patients do not show appropriate electric signals^[10]^, all of our patients were approved to be implanted.

All the implants were successfully accomplished. We did not depend on cardiac anatomy and venous system, but instead we depended on thorax morphology, size and structure. We faced some difficulties to position the lead when tunneling it at the sternal region between proximal and distal incisions; one case due to a great quantity of subcutaneous tissue, patient with a high BMI, and unfortunately this is a condition that has been increasing in our country and currently almost half of Brazilian population are overweight^[11]^. The other one due to a thoracic deformity. In this last one, even after repositioning the lead, the highest defibrillation threshold was achieved. As well as described in the literature^[12]^, our success rate to convert the induced tachyarrhythmia during the implant defibrillator test was quite satisfactory bearing in mind that two out of 18 presented a higher defibrillation threshold than expected, less than 80 Joules. We sought the Technical Consultant Boston Scientific Therapy Systems Support for some help. They informed us that there are a few known causes of high DFTs for SICD devices. The first one would be amiodarone, the second one would be a long time of anesthesia and the third one would be a suboptimal system positioning, and thus if the shock impedance is in the normal range, it is a medical decision whether to wait and try DFT on a different day with medication changes or less anesthesia. As these two patients had been taking amiodarone for a long time, the medicine was withdrawn and the threshold test was redone after 30 days at least. But both patients didn´t return yet to do the new threshold assessment.

Similar to the 3-year follow-up data of EFFORTLESS Registry^[12]^, which is the first great international non-randomized, multicenter registry designed to follow-up and assess the long term clinical and device outcomes from S-ICD, our data show 77.8% of the patients submitted to the intervention for primary prevention (14 patients) and 22.2% for secondary prevention (4 patients). On the other hand, only 22.2% of our patients (4 patients) treated for primary prevention showed EF less than 35% compared with 30.6% of the EFFORTLESS Registry^[12]^. In comparison, our numbers for the disease etiology were also different. Hypertrophic cardiomyopathy was responsible for 27.7% (5 patients) of the indications and ischemic cardiomyopathy for 27.7% (5 patients) compared with 11.7% and 31.1%, respectively, of EFFORTLESS. These differences can be explained, firstly, by the reduced number of our sample and, secondly, although the increasing evidences of indications for primary prevention in ischemic patients with reduced EF and also because this is an initial experience, the trend was choosing indications in younger patients and without the necessity of using a great quantity of negative chronotropic drugs. It is important to notice that four patients had Chagas disease and it is also well known that approximately 60% of these cases, the sinus node is injured, developing a more or less extensive sick sinus syndrome^[13]^ and for that reason we must take care when recommending the S-ICD procedure for chagasic patients.

One patient showed inappropriate shocks a week after the discharge due to oversensing caused by myopotentials and a reintervention to replace the lead was performed because the programming optimization was unsuccessful (in this case, it is very limited). One of the limitations inherent to the device is the presence of inappropriate shocks occurring in up to 8.4% of the cases^[14]^: T-wave oversensing is responsible for 80% of the cases and myopotential oversensing for 5-10%^[10]^.

There was a case of edema in pulse generator pocket with intense pain beginning five days after discharge. The diagnostics of the ultrasound test performed was not really clarifying. A treatment with non-steroidal anti-inflammatory drugs (NSAIDs) and oral therapy with antibiotics over 7 days was prescribed, even without an evidence of infection, since this was the post-operative of a prosthesis implantation and a more bloody surgery although less invasive. This clinical condition was solved with this prescription. Pocket infections in this procedure as well as in the conventional one may occur in 5-10% of the cases, but in such cases the solution with antibiotics therapy without the necessity of withdrawing the system seems to be more probable and can be tried with less risk of endocarditis^[10]^. Two patients demanded medication for pain relief that last five days after the surgery. It is important to notice that it is more bloody procedure than the conventional, so we should consider a special post-surgery care, mainly in those pain sensitive patients.

Initially, the motivation to develop this device was the possibility of treating special cases such as the pediatric population with congenital heart disease, patients without venous access or any other contraindication for a transvenous ICD^[8]^. Many advantages inherent to the S-ICD currently turn it into not only an alternative to the conventional ICD, but the first therapeutical choice for patients at high risk for infection, immunosuppressed patients, patients with prosthetic valves and patients dependent on hemodialysis, since besides the absence of risk for mechanical traumas, such as vascular damages or pneumothorax, the risk for systemic infection is too low^[8]^. Due to its potential benefits, the S-ICD can be considered a feasible alternative for patients with channelopathies, usually young and with a long-life expectancy, as well as for primary and secondary prevention in ischemic and non-ischemic patients.

## CONCLUSION

In our initial experience, the S-ICD implantation showed an acute efficacy to revert induced VF during the procedure performed in pre-selected patients, but it should take into account that in two out of 18 patients demanded high energy delivered shocks in order to achieve this goal. In addition, although it is a less-invasive, simple-accomplishment procedure, it resulted in a more bloody surgery perhaps requiring an operative care different from the conventional. Inappropriate shock by oversensing is a reality in this system, which should be overcome in order not to become a limiting issue for its indication.

**Table:** 

Authors' roles & responsibilities	
FSOS	Substantial contributions to the conception or design of the work; or the acquisition, analysis, or interpretation of data for the work; final approval of the version to be published
VSM	Substantial contributions to the conception or design of the work; or the acquisition, analysis, or interpretation of data for the work; final approval of the version to be published
MCVA	Substantial contributions to the conception or design of the work; or the acquisition, analysis, or interpretation of data for the work; final approval of the version to be published
SCW	Substantial contributions to the conception or design of the work; or the acquisition, analysis, or interpretation of data for the work; final approval of the version to be published
LHBR	Substantial contributions to the conception or design of the work; or the acquisition, analysis, or interpretation of data for the work; final approval of the version to be published
PAGL	Substantial contributions to the conception or design of the work; or the acquisition, analysis, or interpretation of data for the work; final approval of the version to be published
DBM	Substantial contributions to the conception or design of the work; or the acquisition, analysis, or interpretation of data for the work; final approval of the version to be published

## References

[1] Moss A, Zareba W, Hall W, Klein H, Wilber DJ, Cannom DS (2002). Multicenter Automatic Defibrillator Implantation Trial II
Investigators. Prophylactic implantation of a defibrillator in patients with
myocardial infarction and reduced ejection fraction. N Engl J Med.

[2] Bardy GH, Lee KL, Mark DB, Poole JE, Packer DL, Boineau R (2005). Sudden Cardiac Death in Heart Failure Trial (SCD-HeFT)
Investigators. Amiodarone or an implantable cardioverter-defibrillator for
congestive heart failure. N Engl J Med.

[3] Tan VH, Wilton SB, Kuriachan V, Sumner GL, Exner DV (2014). Impact of programming strategies aimed at reducing nonessential
implantable cardioverter defibrillator therapies on mortality: a systematic
review and meta-analysis. Circ Arrhythm Electrophysiol.

[4] Gasparini M, Nisam S (2012). Implantable cardioverter defibrillator harm?. Europace.

[5] Atwater BD, Daubert JP (2012). Implantable cardioverter defibrillators: risks accompany the
life-saving benefits. Heart.

[6] Maisel WH (2007). Transvenous implantable cardioverter-defibrillator leads: the
weakest link. Circulation.

[7] Maisel WH, Kramer DB (2008). Implantable cardioverter-defibrillator lead
performance. Circulation.

[8] Akerström F, Arias MA, Pachón M, Puchol A, Jiménez-López J (2013). Subcutaneous implantable defibrillator, state-of-the art
2013. World J Cardiol.

[9] De Maria E, Olaru A, Cappelli S (2015). The entirely subcutaneous defibrillator (S-ICD): state of the art
and selection of the ideal candidate. Curr Cardiol Rev.

[10] The entirely subcutaneous defibrillator (S-ICD): State of the art and
selection of the ideal candidate.

[11] Costa VEA, Ferolla SM, Reis TO, Rabello RR, Rocha EAV, Couto CMF (2015). Impact of body mass index on outcome in patients undergoing
coronary artery bypass grafting and/or valve replacement
surgery. Rev Bras Cir Cardiovasc.

[12] Lambiase PD, Barr C, Theuns DA, Knops R, Neuzil P, Johansen JB, EFFORTLESS Investigators (2014). Worldwide experience with a totally subcutaneous implantable
defibrillator: early results from the EFFORTLESS S-ICD
registry. Eur Heart J.

[13] Pachon JC (2015). Chronotropic incompetence in Chagas disease: usefulness of dual
sensor pacemaker based on volume minute and accelerometer. Rev Bras Cir Cardiovasc.

[14] Late-breaking clinical trial results announced at heart rhythm 2016:
first long-term results show S-ICD is safe for heart arrhythmia
patients.

